# Assessment of Regional Perfusion and Organ Function: Less and Non-invasive Techniques

**DOI:** 10.3389/fmed.2019.00050

**Published:** 2019-03-22

**Authors:** Wolfgang Huber, Robert Zanner, Gerhard Schneider, Roland Schmid, Tobias Lahmer

**Affiliations:** ^1^Medizinische Klinik und Poliklinik II, Klinikum rechts der Isar, Technische Universität München, München, Germany; ^2^Klinik für Anästhesiologie, Klinikum rechts der Isar, Technische Universität München, München, Germany

**Keywords:** liver function, neuromonitoring, capillary refill time, body surface temperature, near infra-red spectroscopy, hemodynamic monitoring, renal failure, mottling score

## Abstract

Sufficient organ perfusion essentially depends on preserved macro- and micro-circulation. The last two decades brought substantial progress in the development of less and non-invasive monitoring of macro-hemodynamics. However, several recent studies suggest a frequent incoherence of macro- and micro-circulation. Therefore, this review reports on interactions of macro- and micro-circulation as well as on specific regional and micro-circulation. Regarding global micro-circulation the last two decades brought advances in a more systematic approach of clinical examination including capillary refill time, a graded assessment of mottling of the skin and accurate measurement of body surface temperatures. As a kind of link between macro- and microcirculation, a number of biochemical markers can easily be obtained. Among those are central-venous oxygen saturation (S_cv_O_2_), plasma lactate and the difference between central-venous and arterial CO_2_ (cv-a-pCO_2_-gap). These inexpensive markers have become part of clinical routine and guideline recommendations. While their potential to replace parameters of macro-circulation such as cardiac output (CO) is limited, they facilitate the interpretation of the adequacy of CO and other macro-circulatory markers. Furthermore, they give additional hints on micro-circulatory impairment. In addition, a number of more sophisticated technical approaches to quantify and visualize micro-circulation including video-microscopy, laser flowmetry, near-infrared spectroscopy (NIRS), and partial oxygen pressure measurement have been introduced within the last 20 years. These technologies have been extensively used for scientific purposes. Moreover, they have been successfully used for educational purposes and to visualize micro-circulatory disturbances during sepsis and other causes of shock. Despite several studies demonstrating the association of these techniques and parameters with outcome, their practical application still is limited. However, future improvements in automated and “online” diagnosis will help to make these technologies more applicable in clinical routine. This approach is promising with regard to several studies which demonstrated the potential to guide therapy in different types of shock. Finally several organs have specific patterns of circulation related to their special anatomy (liver) or their auto-regulatory capacities (brain, kidney). Therefore, this review also discusses specific issues of monitoring liver, brain, and kidney circulation and function.

## Introduction

### Macro-Circulation

Organ function directly depends on appropriate supply of oxygen and energy. To maintain these prerequisites of cellular integrity and organ function, as well as to remove waste products and toxic metabolites, sufficient circulation, and perfusion are required.

The main determinants of macro-circulation are pressure and flow. Both can be measured directly with a variety of techniques. Both flow and pressure are obviously connected in analogy to Ohm's law of electricity:

Formula of Ohm's Law for Electricity and Its Analogy to Circulation

$$\begin{array}{l} \frac{U}{I}=R\;\frac{MAP-CVP}{CO}=SVR \end{array}$$

U = Voltage; I = Electric flow; R = Resistance; MAP = mean arterial pressure; CVP = central venous pressure; CO = cardiac output.

The electric flow (I) is driven by the difference of potential (voltage U) generated by the energy source, and it is modulated by the resistance within the circuit (R).

Similarly, blood flow (cardiac output CO) is driven by a pressure gradient between mean arterial pressure and central venous pressure (CVP) which is provided by a generator (heart). Obviously CO is modulated by systemic vascular resistance (SVR).

### (In)Coherence of Macro- and Micro-Circulation

Under physiological conditions, macro- and micro-circulation are inter-dependent to a high degree. By contrast, for pathological conditions such as sepsis and other etiologies of shock, the loss of this “coherence” is almost pathognomonic (1, 2). Under these conditions, macro- and micro-circulation are additionally modulated by interactions of inflammation and heterogenic obstruction of the micro-circulation (3).

While CO and MAP are unquestioned cornerstones to provide appropriate perfusion, normal values of both parameters do neither preclude a misbalanced oxygen delivery and demand nor an impaired microcirculation.

## Adequacy of Macro-Circulation: The “Bridge to Micro-Circulation”

### Mixed Venous Oxygen Saturation S_v_O_2_, Central-Venous Oxygen Saturation S_cv_O_2_

To assess the adequacy of global perfusion and appropriate local oxygen supply, a number of biochemical markers have been suggested and included in guideline recommendations (4, 5). Abnormal values of S_cv_O_2_, plasma lactate and cv-a-pCO_2_-gap have been associated with poor outcome in a large number of studies (Table 1). Therapeutic algorithms aiming at normalization of these parameters improved outcome in a number of studies (6, 9), but failed in other trials (10). The use of these parameters is appealing due to their low costs and the easiness of measurement. Their appropriateness to guide therapy is limited in populations with a high prevalence of patients with normal values (10). This might also explain contradictory results of studies using these parameters to guide therapy.

**Table 1:** Markers of adequacy of macro-circulation.

	**Measurement**	**Cut-off**	**Overall potential to guide therapy**	**Comment**
S_v_O_2_	Intermittent Continuous (Oximetry)	70%	++	Requires pulmonary arterial catheter PAC
S_cv_O_2_	Intermittent Continuous [e.g., PreSep® (6); CeVOX (7, 8)]	70%	+++	Interchangeable with S_v_O_2_ for clinical purposes. High values may result from shunting or luxurious perfusion.
Lactate	Intermittent Continuous (e.g., EIRUS®)	2 mmo/L	+++	Part of new definition of septic shock. Increases in lactate levels by ß_2_-stimulation
cv-a-pCO_2_-gap	Intermittent	6 mmHg	++	Requires arterial as well as central venous catheter

In general, normal values of MAP and CO do not preclude pathological values of S_cv_O_2_, plasma lactate, and cv-a-pCO_2_-gap and vice-versa (11–13). Therefore, S_cv_O_2_, plasma lactate and cv-a-pCO_2_-gap are used in a combined approach with CO to reflect the adequacy of CO in a certain clinical context.

Since these parameters provide information in addition to macro-circulation and reflect metabolism, they can be considered as some kind of “bridge to micro-circulation.” Per definition, S_v_O_2_ and S_cv_O_2_ are closely associated with macro-circulation. Applying the Fick equation to O_2_ results in

$$\begin{array}{lll} {\text{S}}_{\text{v}}{\text{O}}_{2} & = & {\text{S}}_{\text{a}}{\text{O}}_{2}-\left(\text{V}{\text{O}}_{2}/\left(\text{C}{\text{O}}^{*}\text{H}{\text{b}}^{*}1.34\right)\right)\\ {\text{S}}_{\text{a}}{\text{O}}_{2} & = & \text{arterialoxygensaturation}\\ \text{V}{\text{O}}_{2} & = & \text{wholebody}{\text{O}}_{2}\text{consumption}\\ \text{Hb} & = & \text{hemoglobin} \end{array}$$

Necessarily, with constant S_a_O_2_, VO_2_, and Hb, decreasing values of CO result in decreases of S_v_O_2_ due to a compensatory increase in the oxygen extraction rate. Normal values for S_(c)v_O_2_ in healthy subjects range from 70 to 75%.

While measurement of S_v_O_2_ requires withdrawal of blood from a pulmonary arterial catheter (PAC) or oximetric measurement with a special PAC, S_cv_O_2_ can be obtained easily from a conventional central venous catheter (CVC) or continuously with a specific oximetric CVC. Although S_cv_O_2_ and S_v_O_2_ may differ due to the slightly different oxygen content of blood returning from the lower and upper half of the body, it is well accepted to replace S_v_O_2_ by S_cv_O_2_ for clinical purposes.

### Lactate

Among the bridges from macro- to micro-circulation plasma lactate is the parameter which is most “down-stream,” i.e., close to microcirculation and cellular metabolism (14). A variety of experimental and clinical studies demonstrated that lactate levels indicating anaerobic metabolism increase in parallel with a decreasing ratio of oxygen utilization divided by oxygen demand. Increasing lactate levels are associated with abnormal oxidative phosphorylation (4). Lactate levels of >2 mmol/L are considered to be abnormal, but also lower cut-offs (>1.5 mmol/L) have been associated with poor outcome in patients with sepsis (15).

At least two trials associated decreasing lactate levels and lactate-guided early-goal directed therapy with improved outcome (9, 16). Finally, lactate has become part of the new definition of septic shock (5).

Based on these findings several recent guidelines recommend lactate measurement every 2 h within the first 8 h and every 8–12 h thereafter after admission with shock (4).

A major advantage of guiding therapy by lactate levels is the easiness of measurement which does not require central-venous access. Similar as for S_v_O_2_ by S_cv_O_2_ devices providing continuous measurement are available (7, 17).

However, it has to be kept in mind that hypoperfusion is not the only reason cause of elevated lactate levels. Impaired liver function and stress can also contribute to increases in lactate levels.

### Central-Venous—Arterial CO_2_ Difference (cv-a-pCO_2_-gap)

Among the parameters used as a bridge to micro-circulation, cv-a-pCO_2_-gap plays an intermediate role between S_cv_O_2_ and lactate. Similar to S_cv_O_2_ the veno-arterial difference in pCO_2_ facilitates interpretation of adequacy of CO and resuscitation. If O_2_-extraction is impaired due to micro-circulatory mal-distribution, S_cv_O_2_ may be normal despite a reduced CO. In this case, a cv-a-pCO_2_-gap >6 mmHg suggests inadequate perfusion, even if S_cv_O_2_ is above 70% (11, 13).

In summary, S_cv_O_2_, plasma lactate and cv-a-pCO_2_-gap have two important roles:

1) When extended hemodynamic monitoring including CO is not available, they can be used as easily measurable indicators of the adequacy of blood flow.

2) If CO is available, pathological values of these markers increase the likelihood of circulatory improvement by increasing CO.

S_v_O_2_: mixed venous oxygen saturation

S_cv_O_2_: central-venous oxygen saturation

cv-a-pCO_2_-gap: central-venous and arterial CO_2_ ().

## Micro-Circulation

While the macro-circulatory interconnections are transparent and easy to be determined, micro-circulation is much more challenging.

Micro-circulation cannot be defined in a clear-cut formula such as Ohm's law. Consequently, assessment of micro-circulation is more complicated due to its dependency on macro-circulation, organ-specific auto-regulatory mechanisms and interactions between certain organs. Furthermore, the technical accessibility to quantify micro-circulation is much more complicated compared to macro-circulation.

Therefore, micro-circulation is assessed based on a plethora of clinical, chemical, and physical surrogates which are frequently restricted to the individual micro-circulation of a single organ.

## Clinical Assessment of Micro-Circulation

The first approach to the assessment of micro-circulation is clinical examination aimed at detection of impaired general and specific circulation (Table 2).

**Table 2:** Structured clinical investigation of micro-circulation.

**Organ**	**Parameter, method**	**Purpose, comment**
Skin	Temperature	Differentiation warm vs. cold shock (18)
Skin	Mottling	Structured assessment of microcirculation (19, 20) of the skin
Skin/nails	Capillary refill time (CRT)	Quantification of capillary perfusion (21)

The most “accessible” organ without any instrumental approach is the skin. Among all organs, skin has the largest weight and contributes about 16% of the body weight, i.e., about 10 kg in a normal weight adult.

A structured assessment of the microcirculation of the skin starts with inspection and palpation aiming at estimation of the surface temperature. In patients with shock this allows for primary classification of “cold” shock and “warm” shock. “Warm shock” is caused by several etiologies of distributive shock including septic, anaphylactic and neurogenic shock.

The etiology of “cold shock” can be hypovolaemic, cardiogenic, or obstructive (pulmonary embolism, pericardial tamponade, pneumo-thorax). “Warm shock” is caused by endogenous or exogenous vasodilators. Most frequently it is due to sepsis. Cold shock typically is mediated by endogenous vasoconstrictors such as nor-adrenaline which is considered as a physiological compensatory mechanism in order to provide and stabilize the perfusion of the most vital organs such as brain, heart and lungs. Most of the other organs including the skin are summarized as “shock organs” that can tolerate markedly reduced perfusion for a certain time.

Regarding the extent of the organ skin, its impaired perfusion is not a regional cosmetic side effect, but has systemic implications for the organism's thermal balance. Abnormal dermal vasoconstriction or vasodilatation results in reduced or increased thermal transfer from the body core to the surface and consecutive changes in the skin temperature (22). Due to the absence of auto-regulatory mechanisms found in the brain, heart and lungs, skin perfusion, and temperature closely reflect the activation of neuro-humoral mechanisms during different forms of shock.

The clinical assessment of the skin temperature should be performed with the investigator's back of the hand, since this part of the hand is most sensitive for temperature. Due to the moderate discriminatory power of this approach, a classification of the temperature as cold, slightly reduced, normal, and warm has been suggested.

To improve the assessment of skin temperature, two approaches can be used:

1. Instrumental measurement of skin temperature
2. Skin-core temperature gradients (SCTG).

The association of cutaneous temperature and cardiovascular function was first described by Hippocrates. The first validation using an instrumental approach was published in 1954 by Felder et al. who demonstrated an association of toe temperature and blood flow measured with a plethysmograph (23). The clinical use of toe temperature measurement to guide vasodilator therapy in shock was described by Ibsen and co-workers (24). Joly and Weil demonstrated a strong association of the toe temperature continuously measured with a probe and cardiac output determined with indicator dilution technique (18). The correlation (*r* = 0.71) was better for toe temperature compared to skin temperature on third finger, deltoid area of the arm, lateral portion of the thigh, and rectal temperature. It was slightly improved when the toe temperature was adjusted to ambient temperature. Furthermore, skin temperature on admission and in particular its changes over time excellently predicted survival. Henning and colleagues demonstrated in 71 patients with acute circulatory failure due to myocardial infarction, sepsis or hypovolaemia that the toe ambient temperature gradient better predicted mortality than cardiac index or arterial pressure (25). A study by Vincent et al. demonstrated that the association of toe-ambient temperature gradient to cardiac output was more pronounced in patients with *cardiogenic* shock compared to *septic* shock (26).

A more recent study investigating the prognostic value of the subjective assessment of peripheral perfusion in critically ill patients showed that central to toe temperature and the skin temperature gradient between the forearm and the index finger were significantly different for patients with and without abnormal peripheral perfusion which was substantially associated to outcome (27). Another recent study demonstrated that toe-to-room and central-to-toe temperature gradients correlated with tissue perfusion and predicted death of multi-organ-failure in septic patients (28).

All of the above-mentioned studies used probes attached to the skin for the measurement of the surface temperatures.

While this allows for intermittent as well as continuous measurement, the attachment of probes also carries several disadvantages: The probes have to be connected to a special monitor which is not ubiquitously available. Furthermore, connection cables maybe disturbing. Finally, continuous attachment of a probe to the skin might alter the temperature at the place of measurement and might cause hygienic problems.

All these problems can be overcome by the use of *non-contact infrared thermometers*. Several recent studies report on comparable predictive capacities of surface temperature measured with non-contact infrared thermometers. While these devices are ubiquitously available and easy to use, the approach of *thermal imagery* is predominantly of scientific and potentially clinical interest. A recent animal study demonstrated significant association of several parameters derived from a non-contact long-wav-infrared camera with MAP, shock-index, paO2, and P/F-ratio (29).

Whereas, other clinical criteria for peripheral perfusion such as mottling of the skin (see below) might fail in patients with colored skin, instrumental measurement of skin temperature is independent of its color.

In addition to the assessment of surface temperature, clinical examination of skin perfusion includes structured static, and dynamic optical examination of the peripheral perfusion.

### Mottling of the Skin

Mottling of the skin has been defined as a patchy skin discoloration that frequently starts around the knees. It is caused by heterogenic constriction of micro-vessels (19). More recently, a structured assessment including a staging (see Table 3) depending on the extent of mottling has been introduced by Ait-Oufella et al. (19). Depending on the extent of the mottled area, this score ranges from 0 to 5.

**Table 3:** Mottling score according to Ait-Oufella (19, 30).

**Mottling score**	**Classification**	**Clinical finding: extent of mottling**
0	No mottling	Normal skin without mottling
1	Modest	Mottling of coin size (center of patella region)
2	Moderate	≤ Upper edge of the knee cap
3	Mild	≤ Middle thigh
4	Severe	≤ Fold of the groin
5	Extremely severe	>Fold of the groin

High inter-observer agreement with a kappa-value of 0.87 has been reported in this study. Baseline mottling-score as well as its changes over time were strongly associated to outcome, whereas mean arterial pressure, CVP, and CI failed to predict 14-days mortality (19). Another study by the same group demonstrated a close association of the mottling score to changes in skin perfusion (30). Based on instrumental investigations with laser Doppler imaging and near-infrared spectroscopy it was shown that in mottled skin areas perfusion as well as tissue oxygenation are reduced (31). The use of the mottling score has been validated in several studies in patients with sepsis (32, 33) as well as liver cirrhosis (34).

### Capillary Refill Time

Capillary refill time (CRT) is defined as the time required for the skin to return to the baseline color after application of a blanching 15s pressure to the distal phalanx of the right and left index (31, 35, 36). The association of CRT with severity of shock was first described more than 70 years ago (20). More recently, CRT has been suggested as a standard in particular for advanced pediatric life support (37) for more than four decades (21). In non-selected critically ill adult patients a CRT >4.5 s was associated with worse outcome. In another study a CRT >5 s was associated with perioperative complications and death after major abdominal surgery (38). Furthermore, a recent study suggests that changes in CRT might be used as targets to stop resuscitation in patients with septic shock (37).

Despite the appealing simplicity of CRT there are several limitations of this parameter: The inter-observer variability has been poorly investigated and resulted in contradictory findings (38–40). Furthermore, a variety of different cut-offs are suggested depending on age and gender of the patients (39, 41, 42).

A recent study demonstrated an association of visceral organ vascular tone with CRT and mottling score, but not with body surface temperature. However, skin temperature was determined in a dichotomous way (warm or cold) by subjective assessment of the examiner, but not with a thermometer or a probe (43).

Normalization of the skin perfusion might be used as a goal for resuscitation, since it occurs earlier during resuscitation than normalization of lactate levels (22, 37, 41).

Finally, a structured combination of clinical parameters of the skin perfusion might improve resuscitation compared to standard algorithms (44) (Table 4).

**Table 4:** Clinical and technical approaches to assess micro-circulation [modified according to Tafner et al. (36)].

**Parameter**	**Method**	**Advantage**	**Limitation**	**Comment**	**References**
Skin temperature	Clinical examination	Easy access	Limited discrimination	First triage of shock	Hippocrates (460–370 B.C.); (27)
	Probe	Quantitative and continuous measurement	Requires disposables Impact of probe on temperature?	Common in pediatrics	(18, 24, 27)
	Infrared thermometer	Quantitative measurement; low costs (no disposables required); hygienic due to non-contact measurement	Intermittent measurement	Feasibility of combination with other non-invasive techniques proven.	(45)
	Infrared camera	Quantitative and continuous measurement	Costs	Additional parameters (e.g., spatial gradient)	(29)
Mottled skin	Clinical examination	Easy to perform. Structured documentation	Colored skin	Easy to train; fast reacting Association with organ perfusion	(19, 30) (43)
Capillary refill time (CRT)	Clinical examination	Easy to perform and quantify	Low sensitivity in children-Variation of normal range with age and gender. Colored skin	Different cut-offs given. Changes in RCT used to stop resuscitation. Association with organ perfusion	(21), (43)
Video-microcopy	Different technical approaches	Direct visualization of micro-circulation	Expenses. Read-out difficult in routine.	Improved read-out recently introduced	(46–48)
Perfusion index	Different technical approaches	Non-invasive	Cost for device; disposables	Different cut-offs given.	(49–51)
StO_2_	Transcutaneous sensors	Continuous measurement	Costs; disposables	Indirect assessment of microvascular flow	(52–54)
NIRS	Near-infrared application usually in the tenar region	Non-invasive; continuous	Costs; measurement impaired by edema and adipositas	Increased information by vascular occlusion test (VOT)	(54–57)

## Technical Assessment of Microcirculation

In addition to the above-mentioned structured clinical approaches to assess peripheral perfusion and body surface temperatures, a number of more sophisticated instrumental techniques have been introduced within the last two decades. Due to their technical properties they are frequently restricted to measurement of microcirculation in certain regions. Extrapolation of the findings in these specific areas to general peripheral perfusion has to be done cautiously. Finally, it has to be kept in mind that these techniques are not widely available and predominantly used for research purposes (36, 58).

### Peripheral Perfusion Index

Based on pulse oximetry and the amount of absorbed infrared light, several commercially available devices provide a “perfusion index” (PI) which is calculated as the ratio of pulsatile blood-flow to non-pulsatile blood-flow (49). Absorption of light with different wavelengths can be measured percutaneously. The absorbance has a pulsatile component resulting from changes in peripheral arterial blood flow and volume, whereas the constant non-pulsatile part of absorption is due to venous blood and extravascular tissues between the light source and the detector.

Baseline values and changes of PI correlate to core-to-toe temperature difference (49). In this study the best cut-off to predict poor tissue perfusion was a PI <1.4. By contrast, a more recent study using a different device demonstrated that a PI >3.5 was associated with detrimental vasodilatation and a higher incidence of hypotension following spinal anesthesia (50). In a study in septic patients a PI <0.2 was associated with poor outcome (51). The main advantage of PI monitoring it is easy to use and inexpensive. On the other hand—as for many of these techniques—the information about micro-circulation is restricted to the investigated area. Furthermore, detection of the signal is impeded by a more pronounced endogenous vasoconstriction or high-dosage vasopressor use.

### Video-Microscopy

Video-microscopy is among the more generally known techniques (58, 59). It is usually performed in the sublingual area (46). Sublingual microcirculatory abnormalities include decreases in the proportion of perfused vessels (PPV), in the total density of small micro-vessels, and in microvascular flow index (MFI) as well as an increased heterogeneity of the micro-perfusion.

These alterations have been associated with hyperlactataemia, vasopressor dependency, organ dysfunction, and mortality (60). Resuscitation and inotropes have been shown to normalize sublingual microcirculatory abnormalities (61). Moreover, reduction in MFI has been identified as an independent risk factor for mortality (62).

The technique has been refined over time. Three generations of devices have been introduced within the last two decades: Orthogonal polarization spectral imaging (OPS), side-stream dark field (SDF; 2nd generation), and incident dark field illumination (IDF; 3rd generation). The 3rd generation technology provides better image resolution and visualization of more capillaries than OPS and SDF-devices (46). Video-microscopy has been used to some extent for educational purposes to make micro-circulatory disturbances more evident. While the educational merits of the technique are unquestioned, its clinical use is limited due to several limitations.

The currently available technologies are time consuming, expensive and require a thorough training as well as a complex analysis of the measurements. Several pitfalls including artifacts induced by compression and saliva have to be considered (46).

The normal ranges of PPV and MFI still are discussed and in part contradictory (22, 63, 64). The large variation of PPV and MFI in healthy volunteers might be in part related to compression artifacts induced by the hand-held video-microscopes (22).

To improve the quality of imaging and to standardize the description of the findings, several scores and consensus recommendations have been established (46, 65).

Despite the use of standardized examination and supporting software the examination remains to be time-consuming (22) and limits a more widespread use.

This might be improved by automated devices. However, at present there are still concerns regarding their reliability. Several studies demonstrated poor agreement of manual with automated measurements of sublingual micro-circulation (66, 67).

If technical development will provide these techniques more readily available for clinical use at a reasonable price, this could be a major step forward to bring microcirculatory parameters into the first line of shock assessment and guidance of therapy (68, 69). However, the additional value of these technologies in addition to thorough clinical examination has been questioned (70).

### Laser Doppler Flowmetry

Laser Doppler Flowmetry (LDF) is based on a confocal technique which analyses vascular density, diameters and flow in arterioles, capillaries and venules (36, 71). While the method has the limitation not to differentiate the flow in these different types of vessels, it has the advantage that it can be used at any region of the skin (36).

### Partial Oxygen Pressure Measurement, Near Infrared Spectroscopy (NIRS)

This method uses transcutaneous electrodes for continuous measurement of oxygen (P_tc_O_2_) and carbon dioxide concentrations in the tissue.

NIRS detects and measures chromophores which are related to the color of molecules. Among those molecules are myoglobin, oxyhemoglobin, and deoxyhemoglobin. Among the parameters derived from NIRS StO_2_ is the most commonly applied one (72). StO_2_ usually is measured in the tenar region where the lack of adipose tissue allows for a close contact with the muscle tissue. The signal detected by NIRS is not directly related to blood flow within a specific vascular bed, since it reflects StO_2_ based on signals from arterioles, capillaries, and venules (36). NIRS signals may be misleading in case of cold extremities (70). The clinical usefulness might be increased by a standardized test with a short term ischemia (vascular occlusion test VOT) which can help to assess the microvascular reserve and oxygen consumption in the tenar muscle (55–57).

In summary, all the above-mentioned techniques provide better insight in micro-circulation than traditional parameters of macro-circulation. Structured clinical assessment of micro-circulation is mandatory in critically ill patients. Future developments will facilitate the use of technical approaches to assess and quantify micro-circulation. This might help to use these parameters to guide resuscitation and to avoid of over-hydration in septic shock (69). However, the superiority of these techniques to meticulous and standardized clinical examination remains to be proven.

## Organ Specific Micro-Circulation

Several organs have specific patterns of circulation related to their special anatomy (liver) or their auto-regulatory capacities (brain, kidney).

Therefore, the following paragraphs will address specific issues of monitoring brain, liver, and kidney circulation and function.

## Monitoring of Microcirculation and Function of the Brain

Although the brain accounts for only 2% of the total body weight in humans, it requires about 20% of the body's blood supply. Brain function depends on the continuous delivery of oxygen and nutrients. Even short interruptions of this supply may lead to decrease of neuronal function and subsequent brain cell death.

## Transcranial Doppler Sonography

Cerebral blood flow (CBF) is of critical importance for cerebral oxygen delivery (CDO_2_). Transcranial Doppler sonography (TCD) can be employed to assess CBF. With TCD, erythrocyte flow velocity is measured in large cerebral arteries using low-frequency ultrasound. Blood flow velocity depends not only on blood flow, but also on the diameter of the arterial vessel, and thus blood flow velocity only provides an estimate of CBF (73).

A flow reduction in cerebral arteries may lead to cerebral ischemia. Unfortunately, a generally valid threshold value cannot be defined. Some authors suggest absolute values of 25 or 30 cm/s (74, 75), others suggest relative reductions ranging from at least 60–80% (76, 77).

TCD has several limitations:

1. Exact quantification of CBF is not possible with TCD, since the exact vessel diameter cannot be determined.
2. Skull bone is largely impermeable for ultrasound waves. Therefore, TCD can only be performed at sites where the bone is rather thin.
3. Reflection of ultrasound (i.e., measured blood flow velocity) depends on the angle between probe and direction of blood flow. Therefore, the result significantly depends on probe position and user experience. This may in part be overcome by the use of transcranial duplex.

Due to these limitations, specific skills and knowledge are a prerequisite in order to obtain reproducible and reliable results with TCD.

## Near-Infrared Spectroscopy

While measuring CBF can give valuable information on cerebral oxygen delivery, it cannot provide information on cerebral oxygen consumption. Therefore, continuous real-time monitoring of cerebral oxygenation using near infrared spectroscopy (NIRS) may provide important therapeutic information. This technique was introduced into clinical practice in the 1980s for the assessment of cerebral oxygenation in preterm infants (78). Similar to pulse oximetry, the technical principle of NIRS is based on differences in the absorption of light by oxygenated and desoxygenated hemoglobin (Hb). Photons emitted at wavelengths in the near-infrared spectrum penetrate scalp, skull bone and brain tissue (79). In contrast to pulse oximetry, NIRS is not based on transluminescence, but analyses reflected photons. The ratio of oxygenated Hb to total Hb, expressed as regional cerebral oxygen saturation (rScO_2_), can be estimated using principles of optical spectrometry. Usually, optodes containing sender and receiver are placed on the patients' forehead (often bilaterally). Measured rScO_2_ values then resemble oxygen saturation in the frontal cortex, an area that is very sensitive to hypoxemia. Since the near-infrared light penetrates extra-cerebral (scalp, skull bone) and intra-cerebral tissues, all of which absorb light, a low cerebral oxygen saturation may be masked by a high extra-cerebral oxygen saturation, which is called “extra-cerebral contamination” (80). This phenomenon can be reduced technically in modern NIRS monitors, but not totally eliminated.

Depending on the algorithm and the number of photon emission wavelengths used, rScO_2_ values may be measured as “relative” or “absolute” values. Although “absolute” values are sometimes claimed to be superior over “relative” values, there is no data yet available supporting this view.

Cerebral blood volume constitutes of ~20% arterial, ~5% capillary, and ~75% venous blood. Therefore, saturation measured by NIRS essentially resembles venous saturation. At room air, rScO_2_ was found to be around 70% in healthy volunteers (81), and around 60% in older general surgery patients (82) as well as in a mixed population of cardiac surgery patients (83). Due to a high individual variance of rScO_2_, it is crucial to obtain baseline readings in all patients. Furthermore, preoperative rScO_2_ values strongly correlate with cardiac performance (84) and are also associated with outcome in cardiac surgery (85). The threshold value of rScO_2_ below which the likelihood of complications increases, is currently under intensive investigation. Furthermore, it is currently unclear for how long or how often rScO_2_ has to go below such a limit in order to negatively influence outcome. Due to the high variability in baseline values, some authors suggest to use relative decreases in rScO_2_ of 20 (86) or 25% (87) as indicators for hypoxemia, rather than absolute values.

Regardless of the threshold used, several studies concerning general and cardiac surgery show, that avoidance of perioperative cerebral desaturation is accompanied by a lower rate of stroke, postoperative cognitive decline, delirium, overall major organ dysfunction and mortality (82, 86, 88–90). Therefore, an acute decrease in rScO_2_ below a threshold needs a thorough work-up of possible causes and consecutive therapy. The algorithm suggested by Denault et al. that includes optimization of head positioning, arterial blood pressure, oxygenation, ventilation, and Hb-concentration has proved effective for this purpose (91).

Today, NIRS monitoring is mainly used in cardiac surgery and surgery of the thoracic aorta. Its use is recommended for correction of congenital heart defects during childhood as well as operations of the aortic arch in children and adults. It may help detect cannula misplacement during cardiopulmonary bypass and identify patients, in which unilateral ante-grade cerebral perfusion is insufficient. Furthermore, NIRS can be helpful to regulate flowrate during unilateral ante-grade cerebral perfusion in order to prevent hypo-perfusion.

NIRS monitoring has also been evaluated outside of cardiac surgery. During carotid endarterectomy, measurement of rScO_2_ would appear to provide valuable information, since hypoxemic stroke strongly contributes to perioperative morbidity and mortality. However, current data are controversial and the use of NIRS during carotid endarterectomy can only be recommended as second line strategy, when monitoring of evoked potentials is not available.

Surgical procedures in beach chair position have been associated with very rare cases of ischemic brain damage in healthy patients, possibly due to cerebral hypo-perfusion. Using NIRS, some studies could identify significant decreases in rScO_2_ when beach chair position was applied, whereas others could not. Due to the very low incidence of cerebral hypoxemia during beach chair position, routinely measuring rScO_2_ seems difficult to justify.

In summary, NIRS monitoring provides a simple, fast, non-invasive, and continuous measurement of rScO_2_ that can be applied on patients of all ages. It may be of particular value when both intra- and extracranial perfusion is altered. Limitations are high costs of single-use optodes, contamination with extracranial signals and reduced sensitivity for detection of focal ischemia due to limited spatial resolution of commercial monitors.

Table 5 summarizes the discussed methods of monitoring brain circulation and function.

**Table 5:** Summary of non-invasive techniques to investigate cerebral circulation.

**Parameter**	**Method**	**Advantages**	**Limitations**
Cerebral blood flow	Transcranial Doppler sonography	Non-invasive Continuous Fast	No exact determination of cerebral blood flow Results dependent on probe position and user experience
Regional cerebral oxygen saturation	Near-infrared spectroscopy	Non-invasive Continuous Applicable to all ages	High costs Possibility of extracranial contamination

## Regional Perfusion of the Liver and Markers of Liver Function

Assessment of liver function is based on markers of excretory function, synthesis, and cellular integrity within the liver. All these criteria are substantially dependent on appropriate perfusion of the liver. Macro-circulation is usually assessed by (Doppler-) ultrasound and CT-(angiography). Direct angiography using arterial, portal-venous or venous access is less frequently required. Furthermore, hepatic hemodynamics can be assessed by catheterization of the liver vein. Use of a balloon catheter and measurement of the occlusion pressure may help to analyze the extent and etiology of portal hypertension.

Impaired hepatic perfusion and metabolism usually affect *excretion, cellular integrity*, and *synthesis* of the liver.

To quantify these three qualities of liver function numerous parameters can be determined routinely. Most of them are considered as *static*, since these parameters except parameters of cellular integrity are slow-reacting and reflect liver function with substantial latency. This applies particularly to the parameters of liver synthesis: The most commonly measured biochemical parameters reflecting liver synthesis cholinesterase ChE and albumin have half-life times of 10 and 20 d, respectively. Fast-reacting parameters such as factor V (half-life time: 4 h) and factor VII (half-life time: 5 h) usually lack a 24/7-availability.

Another draw-back of the *static* parameters is that many of them are not specific for hepatic pathologies. This applies to elevations of bilirubin which can have pre-hepatic (hemolysis), intra-hepatic (cirrhosis, hepatitis), and post-hepatic (biliary obstruction) origin. Similarly, increases in alkaline phosphatase may result from bone pathologies as well as from cholestasis. Furthermore, due to their specific properties and distribution, several enzymes reflect certain hepatic pathologies to a different degree: this applies to glutamate dehydrogenase GLDH and glutamate-oxalacetate-transaminase GOT (= ASAT aspartate-amino-transferase) which reflect particularly peri-central and peri-portal cellular damage, respectively. Both enzymes are not specific for the liver.

Furthermore, the distribution of liver enzymes within the hepatocyte is different: While glutamate-pyruvate-transaminase (= ALAT alanine- amino-transferase) is restricted to the cytoplasm, GOT/ASAT is found in the cytoplasm and mitochondria, and GLDH is restricted to the mitochondria.

To overcome some of these disadvantages, combined scores, and models of liver function have been introduced (Tables 6, 7). These score take into account different qualities of liver function such as excretion and synthesis and combine this information with clinical findings in liver failure (Child-Pugh-score; Table 6) or laboratory data reflecting organ failure associated with severe liver impairment (model of end-stage liver disease (MELD; Table 7).

**Table 6:** Child-Pugh-classification.

**Points**			
**Parameter**	**1 point**	**2 points**	**3 points**
Albumin (g/dl)	>3.5	2.8–3.5	<2.8
Bilirubin (mg/dl)	<2.0	2.0–3.0	>3.0
INR	<1.7	1.7–2.3	>2.3
Ascites	Absent	Easiliy controlled	Large or resisant
Hepatic encephalopathy	Absent	Mild (HE I–II°)	Chronic (III–IV°)
**Child-pugh-Classification**			
	A	B	C
Points	5–6	7–9	10–15

**Table 7:** Model of end-stage liver disease MELD.

MELD-score =	10 ^*^	
	{0.957 Ln (creatinine)	(mg/dl)
	+ 0.378 Ln (bilirubin)	(mg/dl)
	+ 1.12 Ln (INR)	
	+ 0.643}	

Although these classifications and scores have been shown to better reflect severity and prognosis of severe liver impairment compared to single static markers, they can be influenced to a large extent by therapeutic measures such as substitution of plasma and coagulation factors, extracorporeal organ support such as dialysis and liver support, blood transfusion, plasma separation, and clinical interventions such as paracentesis with and without substitution of albumin. One early and important criticism regarding the MELD score was the potential misuse and manipulation of the score by therapeutic interventions influencing serum creatinine, bilirubin, and INR to increase LTX-likelihood. Indeed, it is a general weakness of MELD that all three components can be influenced with and without intent (Table 8).

**Table 8:** Limitations of the model of end-stage liver disease MELD.

	**Inappropriate increase in MELD**	**Inappropriate decrease in MELD**
Creatinine	Inappropriate diuretics Inappropriate initiation of enal replacement therapy RRT (RRT result in MELD-points as for creatinine = 4 mg/dl)	Volume expansion Albumine dialysis
Bilirubin	Hemolysis	Albumine dialysis Other extracorporeal liver support Transfusion Volume expansion
INR	Inappropriate substitution of plasma products (e.g., FFP; PPSB)	Over-substitution of plasma products (e.g., FFP; PPSB)

By contrast, most of the dynamic tests of liver function are not directly influenced by the above-mentioned measures with a potential impact on the association of MELD with liver function.

Interestingly, at least one study demonstrated an independent association of a dynamic liver function test (indocyanine green disappearance rate ICG-PDR; see below) in addition to MELD with survival of patients on the transplantation list (92).

### Dynamic Markers of Liver Function

To overcome the above-mentioned short-comings of static parameters of liver function, a number of *dynamic* tests have been suggested (Table 9). In general, they have in common that they repeatedly or continuously analyze liver function over a short period of time (minutes to hours). Depending on the read-out the results can be obtained online at bedside.

**Table 9:** Indications for dynamic tests of liver function.

Assessment of prognosis of liver failure
Pre partial liver resection assessment of hepatocellular function (“hepatic reserve”)
Allocation for liver transplantation in chronic liver failure
Allocation for liver transplantation in acute liver failure
Adaptation of drug dosage to liver function
Assessment of short term changes in liver function (recovery, deterioration)

Dynamic liver tests are based on excretion, elimination, and metabolism of a test agent that is orally or parenterally applied (Table 10).

**Table 10:** Static and dynamic tests of liver function [modified according to Sakka (93, 94)].

**Static markers of liver function**				**Dynamic tests of liver function**			
**Cellular integrity**	**Excretion**	**Cholestasis**	**Synthesis**	**Elimination**		**Metabolite formation**	
GOT/ASAT	Bilirubin	AP	Albumin	Percutaneous measurement	ICG	Amonipyrine	^14^CO2-exhalation ^13^CO2-exhalation
GPT/ALAT		y-GT	Cholinesterase	Serum measurements	Caffeine	Erythromycin	
GLDH			Factor V		Bromosulf-ophtalein	Methacetin	
LDH			Factor VII		Galactose	Lidocaine	Measurement of serum metabolites

For most of the tests repetitive blood samples are required to measure the kinetics of the agent or its metabolites. Among others, this applies to lidocaine, caffeine, galactose, and bromsulfophtaleine.

After injection of 1 mg/kg lidocaine the agent is N-dealkylated to mono-ethylglycinexylidide (MEGX). To asses liver function, MEGX-concentrations before and in intervals of 15 min after application can be measured, and the MEGX-half-life time can be calculated. MEGX-testing has been used to some extent in clinical studies, and it has been clearly associated with morbidity and mortality (93). Nevertheless, at present the availability within routine laboratories is poor.

The same applies to the caffeine test which is based on measurement of the serum concentrations of the caffeine metabolites paraxanthine, theobromine and theophylline and their ratio to caffeine 4, 8, and 12 h after the oral ingestion of 300 mg of caffeine.

Similarly, the elimination of D-galactose by metabolization to galactose-1-phosphate and further degradation can be quantified (galactose elimination capacity GEC) by repeated blood drawings after infusion of 0.5 g/kg 25% D-galactose solution. Also due to potentially life threatening side effects in case of galactose-intolerance, this test has lost its clinical use. Finally, the elimination of bromsulfopthaleine administered at a dosage of 5 mg/kg can be quantified by drawing blood samples after 30 and 45 min.

While these biochemical metabolic tests all have a pathophysiological rationale and documented associations to morbidity and outcome, their practical use is low due to the need of elaborate non-routine biochemical analyses.

Further limitations of tests based on *oral* application of the test-agent are the frequently impaired gastrointestinal motility and absorption in case of critically ill and patients with liver failure.

As a consequence, in particular two tests with online and bedside availability remain to be more commonly used:

The most widespread method is the indocyanine green (ICG) elimination.

ICG is an infra-red absorbing and fluorescent dye which was originally introduced around WW-II as a dye in photography.

Due to its physical properties and overall low toxicity ICG became wide-spread in critical care in the 1990s. It was used for indicator dilution techniques to derive cardiac output (trans-pulmonary thermo-dilution TPTD) and extravascular lung water EVLW (double-indicator TPTD). Double-indicator TPTD used ice-cold ICG for indicator dilution with two different distribution volumes for the dye (remaining in the vasculature) and the thermal indicator (also diluting in EVLW). Concomitant measurement of the distribution volumes of the thermal indicator and the dye indicator allowed for exact calculation of EVLW. This technique required extra-corporeal or *in vivo* measurement of ICG-concentrations over time. While the time to derive CO and EVLW is short (time for complete transit of the indicator to the site of detection), the prolonged measurement of the ICG elimination curve allows for assessment of the liver function. Double indicator TPTD technique and liver function were combined in at least one commercially available bedside device (COLD: Cardiac Output and Liver Diagnostic; Pulsion Medical Systems, Germany). While the main interest regarding this device might have been advanced hemodynamic monitoring, each measurement necessarily provided dynamic assessment of liver function.

Another bedside available dynamic liver function test which is routinely used to a certain degree in clinical practice is the LiMAX-test (maximum liver function capacity). As for other exhalation tests, the LiMAX test analyses the exhalation of a substrate of a ^13^C or ^14^C-labeled test agent. To overcome the problem of potentially impaired resorption of an orally applied test agent, the LiMAX test quantifies the exhalation of ^13^CO_2_ derived from metabolism of intravenously applied ^13^C-methacetin. The test agent ^13^C-methacetin is metabolized by cytochrome-P450-isoenzyme 1A2 (CYP450 1A2) to acetaminophen (paracetamol) and ^13^CO_2_ which is measured by a breath-test. Similar as for ICG-PDR the results from the LiMAX-test have been associated to outcome and liver-resectability of patients with impaired liver function (95–99).

## Non-Invasive Assessment of Renal Perfusion and Function

Acute kidney injury (AKI) is one of the most common and life-threatening complications which significantly affects morbidity and mortality of intensive care unit patients (100). The most challenging issue in these patients is to identify high-risk patients who should experience early recognition of AKI (100, 101). Overall, diagnosis of AKI is still based on laboratory testing and/or oliguria and its normalization.

However, these more clinical criteria have some limitations. Distinguishing transient AKI from persistent AKI is of clinical relevance, thus stressing the need for criteria to predict its reversibility (102, 103).

One major problem in AKI is that the definition suffered for a long time from a lack of standardized system of identifying and classifying this syndrome. First, the RIFLE criteria (Risk, Injury; Failure, Loss, End stage renal disease) were proposed by the Acute Dialysis Quality Initiative. In the meanwhile, the Kidney Disease Improving Global Outcomes (KDIGO) group defined an unified version of all criteria which now present global consensus (104).

Although urinary analysis and urinary biochemistry have limited clinical utility, the diagnosis of AKI is traditionally based on a rise in serum creatinine and/or fall in urine output (UO). UO is important not only for diagnosis, but also for risk prediction of AKI (105, 106).

Typical causes of AKI in critically ill patients are sepsis, heart failure, hemodynamic instability, hypovolaemia, and exposure to nephrotoxic substances (103). The specific diagnostic workup in individual patients with AKI depends on the clinical context, severity, and duration of AKI, and also on the local availability of the tests. As mentioned above, urinalysis, examination of the urinary sediment, and imaging studies should be performed as a minimum, with additional tests depending on the clinical presentation (107).

Over the last years, basic diagnostics are increasingly being completed by novel biomarkers of AKI. Biomarkers for AKI can be stratified into markers primarily reflecting *glomerular filtration* (i.e., serum cystatin C), *glomerular integrity* (i.e., albuminuria and proteinuria), *tubular stress* [i.e., insulin-like growth factor binding protein 7 (IGFBP-7)], *tubular damage* [i.e., neutrophil gelatinase-associated lipocalin (NGAL), kidney injury molecule-1 (KIM-1)], and *intra-renal inflammation* (i.e., interleukin-18) (108).

The interest in biomarkers is combined with the desire to achieve early diagnosis and detection of renal stress or damage before functional change is evident.

The tubular damage marker “Neutrophil gelatinase associated lipocalin” (NGAL) is one of the most investigated renal biomarker (109). NGAL is typically upregulated in kidney tissue when exposed to nephrotoxic or inflammatory stress, but also released by activated neutrophils with specific forms of the molecule released from the kidney (monomeric) and neutrophils (dimeric) (108, 109). In one analysis of >2,000 critically ill patients, 20% were NGAL-positive without an increase in serum creatinine which can be interpreted as subclinical AKI or false positive results. However, these patients are at great risk of subsequent renal replacement therapy (RRT), longer ICU and hospital stay, and death. Similar findings were observed in emergency department patients (109).

Other molecules such as the kidney injury molecule (KIM-1), tissue inhibitor of metalloproteinases-2 (TIMP-2), and (110) insulin-like growth factor binding protein-7 (IGFBP-7) appears to perform similarly to NGAL, but have not been studied to the extent of NGAL in critically ill patients.

Beyond early diagnosis and risk stratification, these biomarkers may also help to change the definition of AKI in the future and contribute to a better understanding, diagnosis, prevention, and treatment of AKI (110).

In certain circumstances, it may be necessary to use additional tools to diagnose AKI, especially in cases where creatinine and urine values change slowly, are misleading, or cannot be interpreted accurately. This is particularly relevant for critically ill patients where the presence of fluid overload, muscle wasting, sepsis, and reduced effective circulating volume may completely mask the diagnosis of AKI (104).

Renal ultrasonography is useful for evaluating existing structural renal disease and diagnosing obstruction of the urinary collecting system. In detail, the presence of reduced corticomedullary differentiation and decreased kidney size is indicative of underlying CKD (111).

Up to now, there are no good markers of medullary oxygenation as well on how to assess an improvement in medullary oxygenation in AKI (43, 112).

Renal Doppler ultrasound and contrast-enhanced ultrasound are two techniques that may be used at the bedside to estimate renal perfusion and renal cortical microcirculation, respectively (113).

Especially, Doppler-based renal resistive index (RRI) measurement is rapid, non-invasive, and repeatable and may therefore hold promise for monitoring renal function or renal perfusion in critically ill patients (114, 115).

The renal resistive index [RRI = (peak systolic velocity–end diastolic velocity)/peak systolic velocity] consists of the measurement of renal arterial resistances to blood flow detected by echo-color-Doppler system. RRI is reliably correlated with kidney injuries and its severity. In several trials, RRI showed a direct correlation with cardiovascular damage, acute tubular necrosis, and—in septic shock patients—with the prediction of AKI occurrence (114, 115).

Furthermore, depending on the clinical context, patients may require specific immunological tests, including anti-neutrophil cytoplasmic antibody (ANCA), anti-nuclear antibody (ANA), anti-glomerular basement membrane antibody (anti-GBM), and complement component 3 and 4 to rule out immune-mediated diseases (i.e., vasculitis, connective tissue diseases) (104). These investigations should be considered mandatory in patients with AKI presenting primarily with a pulmonary-renal syndrome, hemoptysis, or hemolysis/thrombocytopenia.

Renal biopsies are rarely performed in critically ill patients, mainly due to the perceived risk of bleeding complications and general lack of therapeutic consequences (114). However, a renal biopsy may offer information that is not available through other means and should be considered if underlying parenchymal or glomerular renal disease is suspected (114).

Future techniques try to achieve the ability to rapidly and accurately measure and monitor GFR in real time (115). Optical measurement techniques using minimally invasive or non-invasive techniques that can quantify renal function independent of serum creatinine or urine output are under research (115). In the past few years, some progress has been made in using two-photon excitation fluorescence microscopy to study kidney function (116). It is likely that some of these approaches will enter clinical phase studies in the near future. In the best way, these techniques will enable an earlier diagnosis of AKI and also provide opportunities to improve clinical management, including the use of nephrotoxic substances and appropriate drug dosing (116).

Furthermore, new imaging techniques such as cine phase-contrast magnetic resonance imaging or intra-vital multiphoton studies may be used in AKI detection. However, based on the complexity, financial costs and need for patient transport, it is likely that these methods will remain research tools (117).

## Author Contributions

WH coordinated the review. WH, RZ, and TL drafted the manuscript. GS and RS also participated in the analysis of the data and helped to draft the manuscript.

### Conflict of Interest Statement

WH collaborates with Pulsion Medical Systems SE, Feldkirchen, Germany as member of the Medical Advisory Board. The remaining authors declare that the research was conducted in the absence of any commercial or financial relationships that could be construed as a potential conflict of interest.
